# Correlation of adiposity indices with cardiovascular disease risk factors in healthy adults of Singapore: a cross-sectional study

**DOI:** 10.1186/s40608-016-0114-4

**Published:** 2016-07-07

**Authors:** Xinyan Bi, Siew Ling Tey, Claudia Leong, Rina Quek, Yi Ting Loo, Christiani Jeyakumar Henry

**Affiliations:** Clinical Nutrition Research Centre (CNRC), Singapore Institute for Clinical Sciences (SICS), Agency for Science, Technology and Research (A*STAR), 30 Medical Drive, Singapore, 117609 Singapore; Department of Biochemistry, Yong Loo Lin School of Medicine, National University of Singapore, Singapore, 117599 Singapore

**Keywords:** Adiposity indices, Central adiposity, Waist circumference, General adiposity, Body adiposity index, Body composition, Cardiovascular risk factors, Asian

## Abstract

**Background:**

Obesity has long been highlighted for its association with increased incidence of cardiovascular disease (CVD). Nonetheless, the best adiposity indices to evaluate the CVD risk factors remain contentious and few studies have been performed in Asian populations. In the present study, we compared the association strength of percent body fat (PBF) to indirect anthropometric measures of general adiposity (body mass index (BMI) and body adiposity index (BAI)) and central adiposity (waist circumference (WC), and waist-to-hip ratio (WHR)) for the prediction of CVD risk factors in healthy men and women living in Singapore.

**Methods:**

A total of 125 individuals (63 men and 62 women) took part in this study. PBF was measured by using three different techniques, including bioelectrical impedance analysis (BIA), BOD POD, and dual-energy X-ray absorptiometry (DEXA). Anthropometric measurements (WC, hip circumference (HC), height, and weight), fasting blood glucose (FBG), fasting serum insulin (FSI), and lipid profiles were determined according to standard protocols. Correlations of anthropometric measurements and PBF with CVD risk factors were compared.

**Results:**

Irrespective of the measuring techniques, PBF showed strong positive correlations with FSI, HOMA-IR, TC/HDL, TG/HDL, and LDL/HDL in both genders. While PBF was highly correlated with FBG, SBP, and DBP in females, no significant relationships were observed in males. Amongst the five anthropometric measures of adiposity, BAI was the best predictor for CVD risk factors in female participants (*r* = 0.593 for HOMA-IR, *r* = 0.542 for TG/HDL, *r* = 0.474 for SBP, and *r* = 0.448 for DBP). For males, the combination of WC (*r* = 0.629 for HOMA-IR, and *r* = 0.446 for TG/HDL) and WHR (*r* = 0.352 for SBP, and *r* = 0.366 for DBP) had the best correlation with CVD risk factors.

**Conclusion:**

Measurement of PBF does not outperform the simple anthropometric measurements of obesity, i.e. BAI, WC, and WHR, in the prediction of CVD risk factors in healthy Asian adults. While measures of central adiposity (WC and WHR) tend to show stronger associations with CVD risk factors in males, measures of general adiposity (BAI) seems to be the best predictor in females. The gender differences in the association between adiposity indices and CVD risk factors may relate to different body fat distribution in males and females living in Singapore. These results may find further clinical utility to identify patients with CVD risk factors in a more efficient way.

**Electronic supplementary material:**

The online version of this article (doi:10.1186/s40608-016-0114-4) contains supplementary material, which is available to authorized users.

## Background

Obesity (excess adiposity tissue) is a substantial public health crisis globally with the prevalence increasing rapidly in Asia [1]. Compared with normal weight people, those who are overweight or obese are at greater risk for many diseases, such as cardiovascular disease (CVD), osteoarthritis, diabetes mellitus (DM), and cancers [2, 3]. Despite the clear evidence linking obesity to various poor health outcomes, obesity itself is a complex and heterogeneous condition [4]. Previous study has shown that equally obese subjects with the same amount of total body fat may have markedly different risk factor profiles [5]. The accumulation of intra-abdominal (visceral) adiposity was associated with increased risk of metabolic abnormalities such as insulin resistance and dyslipidemia [5, 6]. However, obese subjects with a normal metabolic risk profile (known as metabolically healthy obese individuals) were generally characterized by low levels of visceral adipose tissue and by subcutaneous obesity [7]. Therefore, the evaluation of total body fat alone is insufficient to distinguish between individuals at high and low risk of CVD. Recent technological advances have made possible the accurate measurement of regional fat compartments using magnetic resonance imaging (MRI) and computed tomography (CT) [8]. These methods may be better predictors of obesity-related health risks, their applications in large epidemiological studies or clinical practice is, nevertheless, not feasible due to the complexity and high costs of the instrumentations.

In contrast, simple-to-use anthropometric indices, e.g. body mass index (BMI; weight in kilograms divided by square of height in meters), waist circumference (WC), hip circumference (HC), waist-to-hip ratio (WHR; ratio of WC to HC), and body adiposity index (BAI; HC divided by height in meters^1.5^, and subtracting 18 from the result), have been widely used as surrogates to correlate with total and subcutaneous body fat volumes for assessing adiposity-related risks [9–14]. Despite years of research, the best measure of adiposity and cut-off values to predict the CVD risk factors have remained contentious. Previous studies have reported that BMI was significantly related with various health outcomes [9]. It has been suggested that BMI was equally effective as WC in identifying individuals at increased risk of CVD [10, 11]. Conversely, some studies reported that WC is a better indicator of CVD risk than BMI and WHR in ethnically diverse groups [12–14], whereas a high WHR has been identified as an increased risk of dyslipidemia, hypertension, CVD and DM compared with BMI [4]. One explanation of these discrepant results could be attributed to the methodological drawbacks of field methods used for anthropometry analysis and the differences in participant characteristics, e.g. age, gender, ethnicity, diet, countries where participants reside [15–17]. Compared to Caucasians, Asians have more visceral adiposity, which is metabolically more adverse, for the same BMI. To date, there have been few studies to examine the relationship between anthropometric adiposity indices to CVD risk factors in Asian populations. Therefore the first objective of this study was to correlate measured anthropometric variables with CVD risk factors, e.g. dyslipidemia, insulin resistance, and blood pressure, in an adult population in Singapore, a South-East Asian country. In addition to the simple anthropometric measures, the commonly used body fat measuring techniques, i.e. dual-energy X-ray absorptiometry (DEXA), BOD POD, and bioelectrical impedance analysis (BIA) were also utilized in this study to estimate the relationship between percent body fat (PBF) and CVD risk factors. Therefore, the secondary objective of this study was to compare the value of simple anthropometric measures of adiposity to advanced fat measuring techniques for the prediction of CVD risk factors.

## Methods

### Study design

This study was limited to cross-sectional analyses of data from participants attending a visit between June 2014 and June 2015. The participants included 125 healthy adults aged 21 to 68 years: 63 males (50.4 %) and 62 females (49.6 %). They were recruited from the general public in Singapore through advertisements and posters that were placed around the National University of Singapore campus, public area and on the Clinical Nutrition Research Centre (CNRC) website. To be eligible, participants were required to be Singaporeans or individuals who have resided in Singapore for a minimum of five years, healthy males and females. Participants were excluded if they were pregnant or diagnosed with any major diseases. Prior to the test day, participants were asked to restrict alcohol and caffeine-containing drinks as well as to refrain themselves from intense physical activity.

### Clinical measures

Participants arrived at the laboratory in the morning after a 10 h overnight fast. Two finger prick capillary blood samples were obtained for determining blood glucose concentration (FBG, mmol/L) using the HemoCue® 201+ RT Glucose analyser (HemoCue Ltd, Dronfield, UK). In addition, a total of 10 mL of venous blood was collected into Vacutainers (Becton Dickinson Diagnostics). Blood samples were separated by centrifugation at 1500 rpm for 10 min at 4 °C within 2 h of being drawn and aliquots were stored at −80 °C until analysis. Fasting serum insulin (FSI, μU/mL) was measured using the immunochemistry analyzer COBAS e411 (Roche, HITACHI, USA). Insulin resistance index HOMA-IR was calculated using FBG and FSI (HOMA-IR = FBG × FSI/22.5).

Fasting lipid parameters including total cholesterol (TC), high density lipoprotein (HDL), low density lipoprotein (LDL), and triglycerides (TG) were measured using chemistry analyzer COBAS c311 (Roche, HITACHI, USA). The ratios of TC/HDL, TG/HDL, and LDL/HDL were calculated from the standard lipid profile. Systolic blood pressure (SBP) and diastolic blood pressure (DBP) were measured with an Omron blood pressure monitor (model HEM-907). The measurements were done in duplicate and readings were averaged.

Standing height was measured to the nearest millimetre with a stadiometer. Body weight and composition were measured to the nearest 0.1 kg by using an 8-electrode BIA device (Tanita BC-418, Tokyo, Japan). Participants were weighed in light clothing without footwear. PBF was measured by using Bod Pod™® Body Composition Tracking System (Cosmed, Rome, Italy; software version 5.2.0) and DEXA (QDR 4500A, fan-beam densitometer, software version 8.21; Hologic, Waltham, USA). WC and HC were measured with an anthropometric measuring tape while the participants were dressed in light clothing. Measurements were taken according to the ISAK International Standards for Anthropometric Assessment guidelines [18]. WC was measured at the minimum circumference between the iliac crest and the rib cage. HC was measured at the maximum protuberance of the buttocks. All of these anthropometric measurements were done in duplicate and readings were averaged.

The body mass index (BMI) was calculated using weight (kg) divided by the height squared (m^2^) whereas the body adipose index (BAI) was calculated using HC and height (BAI = (HC in centimetres)/(height in meters)^1.5^ - 18).

### Statistical analysis

Baseline characteristics of the participants were presented as arithmetic means ± SDs. Baseline characteristics was compared between the two gender groups by using independent-samples *t* tests. Pearson’s correlations were used to investigate the associations between adiposity indices and various CVD risk factors. Linear regression models, adjusted for age, were used to examine the adjusted associations. All statistical analyses were performed using Stata 11.1 (StataCorp, College Station, Tex, USA). Two sided *p* < 0.05 was considered statistically significant in all cases.

## Results

The clinical characteristics of the study population are shown in Table 1. Almost equal men (50.4 %) and women (49.6 %) with similar average age took part in the study. Amongst the anthropometric measurements, males had significantly higher height, weight, BMI, WC, and WHR than females, while females had significantly higher PBF and BAI than males. With the exception of HDL (1.5 ± 0.3 for males and 1.7 ± 0.4 for females, *p* = 0.001), systolic blood pressure (117.7 ± 10.4 for males and 105.3 ± 17.8 for females, *p* < 0.001) and diastolic blood pressure (67.7 ± 8.6 for males and 64.4 ± 9.5 for females, *p* = 0.046), all of the cardiovascular traits did not differ significantly between males and females.

**Table 1:** Clinical characteristics of the study population

	Total (*n* = 125)	Male (*n* = 63)	Female (*n* = 62)	*p* value
Age (y)	31.4 ± 12.2	30.6 ± 11.6	32.2 ± 12.8	0.452
Height (cm)	166.5 ± 9.0	172.4 ± 6.8	160.6 ± 6.9	<0.001
Weight (kg)	63.3 ± 13.9	70.3 ± 11.9	56.2 ± 12.2	<0.001
BMI (kg/m^2^)	22.7 ± 3.9	23.6 ± 3.8	21.7 ± 3.9	0.006
WC (cm)	73.5 ± 10.4	78.0 ± 9.4	68.9 ± 9.4	<0.001
HC (cm)	90.8 ± 7.5	91.7 ± 7.1	89.9 ± 7.8	0.196
WHR	0.81 ± 0.07	0.85 ± 0.06	0.76 ± 0.06	<0.001
PBF^a^	24.1 ± 8.4	18.8 ± 5.8	29.5 ± 7.2	<0.001
PBF^b^	25.2 ± 9.4	20.1 ± 7.8	30.4 ± 7.9	<0.001
PBF^c^	29.2 ± 8.3	23.5 ± 5.6	35.2 ± 6.1	<0.001
BAI	24.4 ± 4.1	22.6 ± 3.5	26.2 ± 3.8	<0.001
SBP (mm Hg)	111.5 ± 15.8	117.7 ± 10.4	105.3 ± 17.8	<0.001
DBP (mm Hg)	66.1 ± 9.2	67.7 ± 8.6	64.4 ± 9.5	0.046
FBG (mmol/L)	4.6 ± 0.5	4.6 ± 0.5	4.5 ± 0.5	0.313
FSI (mU/L)	8.3 ± 5.3	7.9 ± 5.6	8.8 ± 4.9	0.349
HOMA-IR	1.7 ± 1.2	1.6 ± 1.3	1.8 ± 1.2	0.474
TG (mmol/L)	0.86 ± 0.44	0.89 ± 0.40	0.83 ± 0.49	0.472
TC (mmol/L)	5.0 ± 1.4	5.0 ± 1.8	5.1 ± 0.9	0.666
HDL (mmol/L)	1.6 ± 0.4	1.5 ± 0.3	1.7 ± 0.4	0.001
LDL (mmol/L)	3.1 ± 1.2	3.1 ± 1.6	3.0 ± 0.8	0.777
TC/HDL	3.3 ± 1.1	3.5 ± 1.2	3.2 ± 1.0	0.056
TG/HDL	0.62 ± 0.51	0.67 ± 0.45	0.57 ± 0.57	0.277
LDL/HDL	2.1 ± 0.9	2.2 ± 1.0	1.9 ± 0.8	0.051

Values are expressed as mean ± SD. *p* values indicate results of independent samples *t* tests between gender groups

PBF was measured by ^a^BIA, ^b^BOD POD, and ^c^DEXA, respectively

Abbreviations: *BAI* body adiposity index, *BIA* bioelectrical impedance analysis, *BMI* body mass index, *DBP* diastolic blood pressure, *DEXA* dual-energy X-ray absorptiometry, *FBG* fasting blood glucose, *FSI* fasting serum insulin, *HC* hip circumference, *HDL* high density lipoprotein, *HOMA-IR* homeostasis model assessment of insulin resistance, *LDL* low density lipoprotein, *SBP* systolic blood pressure, *TC* total cholesterol, *TG* triglycerides, *WC* waist circumference, *WHR* waist-to-hip ratio

Results obtained from Pearson’s correlation were comparable to those obtained from linear regression models adjusting for age, e.g. statistically significant results remained largely unchanged while non-significant results remained the same (data not shown). Table 2 shows the associations between various anthropometric adiposity indices and CVD risk factors in male participants. All five adiposity indices (i.e. BMI, WC, HC, WHR, and BAI) were significantly correlated with FSI, HOMA-IR, TG, and TG/HDL. Comparison of these adiposity indices in the strength of their correlations with CVD variables revealed that WC had the best correlation with HOMA-IR (*r* = 0.629), TC/HDL (*r* = 0.315), and TG/HDL (*r* = 0.446). On the other hand, WHR was the only adiposity index showing significantly association with SBP (*r* = 0.352) and DBP (*r* = 0.366) in males.

**Table 2:** Pearson’s correlation coefficients of anthropometric measurements with cardiovascular risk factors for male participants (*n* = 63)

	BMI (kg/m^2^)	WC (cm)	HC (cm)	WHR	BAI
FBG (mmol/L)	0.097	0.145	0.108	0.124	0.048
FSI (mU/L)	0.529**	0.628**	0.544**	0.471**	0.403**
HOMA-IR	0.534**	0.629**	0.548**	0.464**	0.408**
TG (mmol/L)	0.411**	0.412**	0.407**	0.264*	0.424**
HDL (mmol/L)	−0.267*	−0.374**	−0.297*	−0.330*	−0.174
LDL (mmol/L)	0.022	0.049	0.071	0.007	0.048
TC (mmol/L)	−0.005	−0.001	0.029	−0.034	0.038
TC/HDL	0.238	0.315*	0.308*	0.210	0.231
TG/HDL	0.428**	0.446**	0.410**	0.322*	0.445**
LDL/HDL	0.209	0.290*	0.292*	0.186	0.200
SBP (mm Hg)	0.138	0.228	0.060	0.352*	0.066
DBP (mm Hg)	0.076	0.200	−0.001	0.366**	−0.007

*Correlation is significant at *p* < 0.05

**Correlation is significant at *p* < 0.005

Abbreviations: *BAI* body adiposity index, *BMI* body mass index, *DBP* diastolic blood pressure, *FBG* fasting blood glucose, *FSI* fasting serum insulin, *HC* hip circumference, *HDL* high density lipoprotein, *HOMA-IR* homeostasis model assessment of insulin resistance, *LDL* low density lipoprotein, *SBP* systolic blood pressure, *TC* total cholesterol, *TG* triglycerides, *WC* waist circumference, *WHR* waist-to-hip ratio

The relationships of anthropometric adiposity indices with CVD risk factors were found to be gender dependent. As observed in Table 3, BMI, WC, HC, WHR, and BAI were significantly correlated with all CVD risk factors, except for LDL and TC in female participants. Amongst them, the Pearson’s correlation coefficients of BAI with HOMA-IR (*r* = 0.593), TC/HDL (*r* = 0.522), TG/HDL (*r* = 0.542), LDL/HDL (*r* = 0.497), SBP (*r* = 0.474), and DBP (*r* = 0.448) were the strongest.

**Table 3:** Pearson’s correlation coefficients of anthropometric measurements with cardiovascular risk factors for female participants (*n* = 62)

	BMI (kg/m^2^)	WC (cm)	HC (cm)	WHR	BAI
FBG (mmol/L)	0.444**	0.454**	0.341*	0.420**	0.452**
FSI (mU/L)	0.546**	0.549**	0.466**	0.438**	0.563**
HOMA-IR	0.559**	0.561**	0.465**	0.460**	0.593**
TG (mmol/L)	0.384**	0.417**	0.289*	0.406**	0.541**
HDL (mmol/L)	−0.419**	−0.398**	−0.228	−0.442**	−0.509**
LDL (mmol/L)	0.177	0.176	0.214	0.074	0.202
TC (mmol/L)	0.071	0.085	0.161	−0.028	0.075
TC/HDL	0.419**	0.425**	0.304*	0.405**	0.522**
TG/HDL	0.401**	0.422**	0.272*	0.432**	0.542**
LDL/HDL	0.399**	0.402**	0.294*	0.376**	0.497**
SBP (mm Hg)	0.403**	0.385**	0.283*	0.369**	0.474**
DBP (mm Hg)	0.452**	0.433**	0.363**	0.346*	0.448**

*Correlation is significant at *p* < 0.05

**Correlation is significant at *p* < 0.005

Abbreviations: *BAI* body adiposity index, *BMI* body mass index, *DBP* diastolic blood pressure, *FBG* fasting blood glucose, *FSI* fasting serum insulin, *HC* hip circumference, *HDL* high density lipoprotein, *HOMA-IR* homeostasis model assessment of insulin resistance, *LDL* low density lipoprotein, *SBP* systolic blood pressure, *TC* total cholesterol, *TG* triglycerides, *WC* waist circumference, *WHR* waist-to-hip ratio

As a direct measurement of body adiposity, PBF was assessed in the strength of its associations with CVD risk factors as presented in Table 4 (males and females separately) and Additional file 1: Table S1 (all participants). Table 4 shows that PBF, measured by three different techniques, shared a comparable pattern of correlation for both genders. We found that PBF was significantly correlated with all CVD risk factors except for TC in female participants. No significant correlations were observed between PBF and FBG, LDL, TC, SBP, and DBP in males.

**Table 4:** Pearson’s correlation coefficients of PBF with cardiovascular risk factors for male (*n* = 63) and female (*n* = 62) participants

	PBF^a^		PBF^b^		PBF^c^	
	Male	Female	Male	Female	Male	Female
FBG (mmol/L)	0.087	0.482**	0.015	0.506**	0.041	0.462**
FSI (mU/L)	0.595**	0.589**	0.547**	0.536**	0.524**	0.542**
HOMA-IR	0.581**	0.601**	0.526**	0.583**	0.504**	0.574**
TG (mmol/L)	0.435**	0.426**	0.327*	0.482**	0.300*	0.442**
HDL (mmol/L)	−0.462**	−0.435**	−0.400**	−0.359**	−0.356*	−0.352*
LDL (mmol/L)	0.118	0.255*	0.257*	0.370**	0.195	0.311*
TC (mmol/L)	0.053	0.151	0.191	0.297*	0.138	0.248
TC/HDL	0.432**	0.488**	0.500**	0.519**	0.437**	0.474**
TG/HDL	0.490**	0.440**	0.401**	0.438**	0.361**	0.401**
LDL/HDL	0.405**	0.466**	0.484**	0.501**	0.423**	0.451**
SBP (mm Hg)	0.130	0.452**	0.085	0.462**	0.081	0.408**
DBP (mm Hg)	0.146	0.491**	0.139	0.517**	0.100	0.471**

*Correlation is significant at *p* < 0.05

**Correlation is significant at *p* < 0.005

PBF was measured by ^a^BIA, ^b^BOD POD, and ^c^DEXA, respectively

Abbreviations: *BIA* bioelectrical impedance analysis, *DBP* diastolic blood pressure, *DEXA* dual-energy X-ray absorptiometry, *FBG* fasting blood glucose, *FSI* fasting serum insulin, *HDL* high density lipoprotein, *HOMA-IR* homeostasis model assessment of insulin resistance, *LDL* low density lipoprotein, *PBF* percent body fat, *SBP* systolic blood pressure, *TC* total cholesterol, *TG* triglycerides

**Fig. 1:**
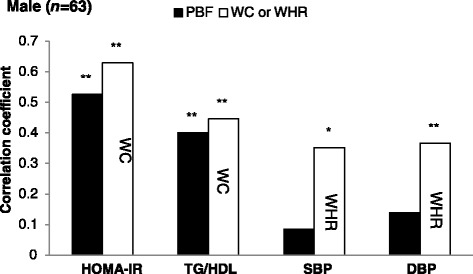
Comparison of the correlation strength of PBF derived from BOD POD and WC or WHR with the CVD risk factors, e.g. HOMA-IR, TG/HDL, SBP, and DBP, in male participants (*n* = 63)

**Fig. 2:**
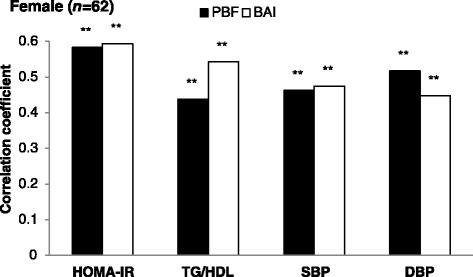
Comparison of the correlation strength of PBF derived from BOD POD and BAI with the CVD risk factors, e.g. HOMA-IR, TG/HDL, SBP, and DBP, in female participants (*n* = 62)

We compared the correlation strength of PBF derived from BOD POD and anthropometric adiposity indices with the CVD risk factors in the current study population (Fig. 1 for males and Fig. 2 for females). Figure 1 shows that WC outperformed PBF in the strength of its correlation with HOMA-IR and TG/HDL and WHR had significant correlations with SBP and DBP, but not PBF in male participants. Figure 2 shows that BAI had a comparable correlation with CVD risk factors as PBF in female participants. The comparison of five anthropometric adiposity indices in their strength of correlations with CVD risk factors are shown in Additional file 1: Figures S1 and S2.

## Discussion

Although the advanced instrumentations, such as DEXA and MRI, have been employed to measure PBF accurately, they are not readily available in most epidemiological studies due to the high cost and complexity. In contrast, anthropometric obesity measurements that reflect body fat are of importance in clinical settings because they require less expense and expertise. Although several studies have successfully included the anthropometric indices of body adiposity in the assessment of CVD risk factors [19–22], the best measure to predict these risk factors remained contentious, especially in Asians. Previous studies suggested that central obesity measures were more strongly associated with CVD risk factors compared with general obesity measures [21]. This is probably because central obesity is associated with systemic inflammation which directly contributes to CVD risk [23]. Conversely, some other studies, like ours, reported that the association between general obesity and CVD was similar to the association between central obesity measures and CVD [24, 25]. We found that both general obesity (BMI) and central obesity (WC) correlated significantly more with seven of the CVD risk factors (FBG, FSI, HOMA-IR, TG, TC/HDL, TG/HDL, LDL/HDL) than HC when all participants were considered together. Moreover, WHR correlated the best with HDL, SBP, and DBP (Additional file 1: Table S1).

BAI was recently proposed to offer a simple-to-use tool to estimate body adiposity [26]. The assessment of BAI’s association with CVD risk factors has been done in various populations with different ethnicities showing that BAI was inferior to other anthropometric measures such as BMI and WC as a predictor of CVD risk factors [20, 27–30]. This present study shows that BAI and PBF shared a comparable correlation pattern, suggesting that BAI, as a measure of overall adiposity, seemed to correlate with PBF better than other anthropometric indices. However, there was no case in which BAI and PBF outperformed these indices with regards to their correlation with several CVD risk factors including HDL, TC/HDL, LDL/HDL, SBP, and DBP in the gender-pooled analyses. This is consistent with prior investigation of body composition and health risk showing that PBF did not perform better than BMI or WC in predicting metabolic risks [31]. Although BAI is related to PBF, it may not be a good indicator of CVD risks as observed in the current population. A circumspect approach may be to use WC and WHR in routine clinical assessment since they are easy to measure and less prone to measurement and calculation errors [32].

An intriguing result of this study was the pronounced gender difference in the correlations of body adiposity measures with CVD risk factors. For male participants, central obesity (WC and WHR) was more strongly associated with CVD risk factors, whereas BAI appeared to be a better predictor of CVD risk factors in female participants (Tables 2 and 3). This may be attributed to the different distribution of adipose tissue in males and females. Compared to men who store more fat in the visceral depot, women store them in the gluteal-femoral region [33, 34]. Therefore, women had less visceral fat despite having a higher total body fat [35]. While some epidemiological studies have concluded that visceral adipose tissue was a stronger correlate of CVD [36, 37], others have argued that subcutaneous adipose tissue may have protective effects [38, 39]. It has thus been hypothesized that the different fat distribution in males and females may contribute to the different correlation of anthropometric obesity indices with CVD risk factors.

## Conclusions

In conclusion, our study reports that several simple anthropometric measurements of obesity, i.e. BAI, WC, and WHR, outperform the measurement of PBF in the prediction of CVD risk factors in healthy males and females living in Singapore. Our data suggests that the correlations between obesity indices and CVD risks are gender-dependent. While measures of central adiposity show stronger associations with CVD risk factors in males, measures of general adiposity show stronger associations in females.

Our study has several limitations that warrant further investigation. This is a cross sectional study and thus causal inferences cannot be drawn. Moreover, the sample size of this study is small. Future studies should be conducted with larger sample sizes. Additionally, although HOMA-IR has become a widely used clinical and epidemiological tool, it is not a direct measurement of insulin resistance. Therefore, the use of HOMA-IR to assess insulin resistance may have potential problems and needs further validation. Despite these limitations, the results of our study provide evidence of the linkage between simple anthropometric measurements and the purported associations between adiposity and health markers.

## Abbreviations

BAI, body adiposity index; BIA, bioelectrical impedance analysis; BMI, body mass index; CVD, cardiovascular disease; CT, computed tomography; DBP, diastolic blood pressure; DEXA, dual-energy X-ray absorptiometry; DM, diabetes mellitus; FBG, fasting blood glucose; FSI, fasting serum insulin; HC, hip circumference; HDL, high density lipoprotein; HOMA-IR, homeostasis model assessment of insulin resistance; LDL, low density lipoprotein; MRI, magnetic resonance imaging; PBF, percent body fat; SBP, systolic blood pressure; TC, total cholesterol; TG, triglycerides; WC, waist circumference; WHR, waist-to-hip ratio

## Additional file

Additional file 1: Supporting information. **Table S1.** Correlation coefficients of anthropometric measurements and PBF with cardiovascular risk factors for all participants. **Figure S1.** Correlation coefficients between different anthropometric measurements and CVD risk factors in female participants. **Figure S2.** Correlation coefficients between different anthropometric measurements and CVD risk factors in male participants. (DOCX 25 kb)
