# Supplementary material for: Correlation of adiposity indices with cardiovascular disease risk factors in healthy adults of Singapore: a cross-sectional study
Source: BMC Obes. 2016 Jul 7;3:33. doi: 10.1186/s40608-016-0114-4 (PMC4936162; doi:10.1186/s40608-016-0114-4)
Supplement: Additional file 1: — Supporting information. Table S1. Correlation coefficients of anthropometric measurements and PBF with cardiovascular risk factors for all participants. Figure S1. Correlation coefficients between different anthropometric measurements and CVD risk factors in female participants. Figure S2. Correlation coefficients between different anthropometric measurements and CVD risk factors in male participants. (DOCX 25 kb) [file 40608_2016_114_MOESM1_ESM.docx]

**Supporting Information**

Correlation of Adiposity Indices with Cardiovascular Disease Risk Factors in Healthy Adults of Singapore: a Cross-Sectional Study

Xinyan Bi,^1^ Siew Ling Tey,^1^ Claudia Leong,^1^ Rina Quek,^1^ Yi Ting Loo,^1^ Christiani Jeyakumar Henry^1,2^

^1^ Clinical Nutrition Research Centre (CNRC)

Singapore Institute for Clinical Sciences (SICS)

Agency for Science, Technology and Research (A*STAR)

30 Medical Drive, Singapore 117609

^2^ Department of Biochemistry

Yong Loo Lin School of Medicine

National University of Singapore, Singapore 117599

X Bi: [bi_xinyan@sics.a-star.edu.sg](mailto:bi_xinyan@sics.a-star.edu.sg)

SL Tey: [siewling_tey@sics.a-star.edu.sg](mailto:siewling_tey@sics.a-star.edu.sg)

C Leong: [claudia_leong@sics.a-star.edu.sg](mailto:claudia_leong@sics.a-star.edu.sg)

R Quek: [rina_quek@sics.a-star.edu.sg](mailto:rina_quek@sics.a-star.edu.sg)

YT Loo: loo_yi_ting@sics.a-star.edu.sg

Correspondence to Professor Christiani Jeyakumar Henry

Tel: +65 64070793

Email: [jeya_henry@sics.a-star.edu.sg](mailto:jeya_henry@sics.a-star.edu.sg)

When the results of all the participants were analysed together, we found that BMI, WC, HC, and WHR were significantly correlated with all CVD risk factors, except for LDL and TC (Table S1). Comparison of these anthropometric measurements in the strength of their correlations with CVD variables revealed that BMI and WC were more strongly correlated to FBG, FSI, HOMA-IR, TG, TC/HDL, TG/HDL, and LDL/HDL than HC and WHR. However, WHR outperformed other anthropometric measurements in the strength of its association with HDL, SBP, and DBP. Table S1 shows that PBF, irrespective of the measuring techniques, and BAI shared a comparable pattern of correlation. Both PBF and BAI showed significant associations with FSI, HOMA-IR, TG, TC/HDL, TG/HDL, and LDL/HDL whereas there were lack of correlations with HDL, SBP, and DBP. Unlike PBF, BAI showed a significant correlation with FBG. It should be noted that of amongst all the adiposity indices, only BOD POD derived PBF was significantly correlated with LDL and TC.

Table S1. Correlation coefficients of anthropometric measurements and PBF with cardiovascular risk factors for all participants (*n*=125).

|  | BMI (kg/m^2^) | WC (cm) | HC (cm) | WHR | BAI | PBF^a^ | PBF^b^ | PBF^c^ |
| --- | --- | --- | --- | --- | --- | --- | --- | --- |
| FBG (mmol/L) | 0.283** | 0.309** | 0.235* | 0.277** | 0.176* | 0.158 | 0.152 | 0.092 |
| FSI (mU/L) | 0.496** | 0.491** | 0.487** | 0.307** | 0.463** | 0.501** | 0.496** | 0.425** |
| HOMA-IR | 0.510** | 0.505** | 0.492** | 0.325** | 0.474** | 0.492** | 0.497** | 0.418** |
| TG (mmol/L) | 0.398** | 0.398** | 0.343** | 0.310** | 0.408** | 0.289** | 0.307** | 0.225* |
| HDL (mmol/L) | -0.397** | -0.465** | -0.280** | -0.481** | -0.168 | -0.130 | -0.131 | -0.023 |
| LDL (mmol/L) | 0.077 | 0.094 | 0.117 | 0.045 | 0.069 | 0.095 | 0.213* | 0.139 |
| TC (mmol/L) | 0.011 | 0.009 | 0.067 | -0.045 | 0.058 | 0.085 | 0.199* | 0.151 |
| TC/HDL | 0.345** | 0.396** | 0.316** | 0.336** | 0.242* | 0.231* | 0.322** | 0.205* |
| TG/HDL | 0.420** | 0.427** | 0.337** | 0.361** | 0.400** | 0.290** | 0.295** | 0.205* |
| LDL/HDL | 0.317** | 0.372** | 0.301** | 0.313** | 0.208* | 0.206* | 0.303** | 0.186* |
| SBP (mm Hg) | 0.362** | 0.434** | 0.229* | 0.497** | 0.088 | -0.010 | 0.022 | -0.082 |
| DBP (mm Hg) | 0.306** | 0.362** | 0.215* | 0.386** | 0.132 | 0.146 | 0.179* | 0.103 |

*Correlation is significant at *p* < 0.05.

**Correlation is significant at *p* < 0.005.

PBF were measured by ^a^BIA, ^b^BOD POD, and ^c^DEXA, respectively.

Abbreviations: BMI, body mass index; WC, waist circumference; HC, hip circumference; WHR, waist-to-hip ratio; BAI, body adiposity index; SBP, systolic blood pressure; DBP, diastolic blood pressure; FBG, fasting blood glucose; FSI, fasting serum insulin; HOMA-IR, homeostasis model assessment of insulin resistance; TG, triglycerides; TC, total cholesterol; HDL, high density lipoprotein; LDL, low density lipoprotein; BIA, bioelectrical impedance analysis; DEXA, dual-energy X-ray absorptiometry.

Figure S1 shows that BAI outperformed other four anthropometric adiposity indices in the strength of its correlation with ten of the CVD risk factors for female participants. It is noteworthy that correlations with FBG were similar for BAI and WC (*r* = 0.452 versus *r* = 0.454; *p* < 0.001). Similarly, the differences in the correlation coefficient between BAI and DBP (*r* = 0.448) and that between BMI and DBP (*r* = 0.452) were small. Figure S2 illustrates the findings for male participants. It was observed that WHR was the only index correlated with SBP and DBP. WC was more strongly correlated with seven out of the nine CVD risk factor variables, including FSI, HOMA-IR, TG, HDL, TC/HDL, TG/HDL, and LDL/HDL, compared to other adiposity indices in males. Among them, WC and BAI were similar in the strength of their correlations with TG (*r* = 0.412 and 0.424, respectively). In addition, WC and HC were similarly associated with LDL/HDL (*r* = 0.290 and 0.292, respectively). Figure S1 and S2 also show that no anthropometric adiposity indices were correlated with LDL and TC in both males and females. Moreover, no significant association was found between FBG and any of the adiposity indices in males (Figure S2).

Figure S1. Correlation coefficients between different anthropometric measurements and CVD risk factors in female participants. All are significant at the level of <0.05.

Figure S2. Correlation coefficients between different anthropometric measurements and CVD risk factors in male participants. All are significant at the level of <0.05.
